# Supplementary material for: Three-dimensional surface topography of graphene by divergent beam electron diffraction
Source: Nat Commun. 2017 Feb 14;8:14440. doi: 10.1038/ncomms14440 (PMC5316882; doi:10.1038/ncomms14440)
Supplement: Supplementary Information — Supplementary Figures and Supplementary Note. [file ncomms14440-s1.pdf]

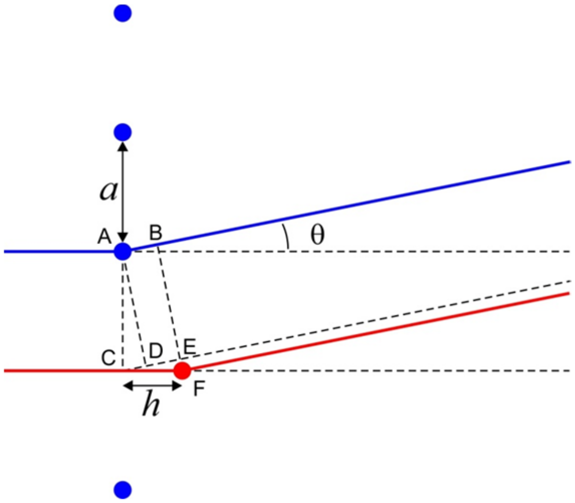

Supplementary Figure 1. Phase change at an out-of-plane ripple. We consider scattering off two atoms at different *z*-positions in an out-of-plane ripple, atoms A and F. The difference in the optical path length between the wave scattered off atom A and atom F equals: $\Delta s = \mathrm{CF} - \mathrm{AB}$, where $\mathrm{AB} = \mathrm{CE} - \mathrm{CD} = h\cos\vartheta - a\sin\vartheta$, so that $\Delta s = h - h\cos\vartheta + a\sin\vartheta = h(1-\cos\vartheta) + a\sin\vartheta$, and the phase shift equals $\Delta\varphi = \frac{2\pi}{\lambda}\Delta s = \frac{2\pi}{\lambda}h(1-\cos\vartheta) + \frac{2\pi}{\lambda}a\sin\vartheta$. Without a ripple, the phase shift is given by $\Delta\varphi = \frac{2\pi}{\lambda}a\sin\vartheta$, which is the phase shift gained by conventional diffraction on periodical lattice. When a ripple is present $h \neq 0$ and the phase shift has additional term $\frac{2\pi}{\lambda}h(1-\cos\vartheta)$, which is pronounced at higher scattering angles.

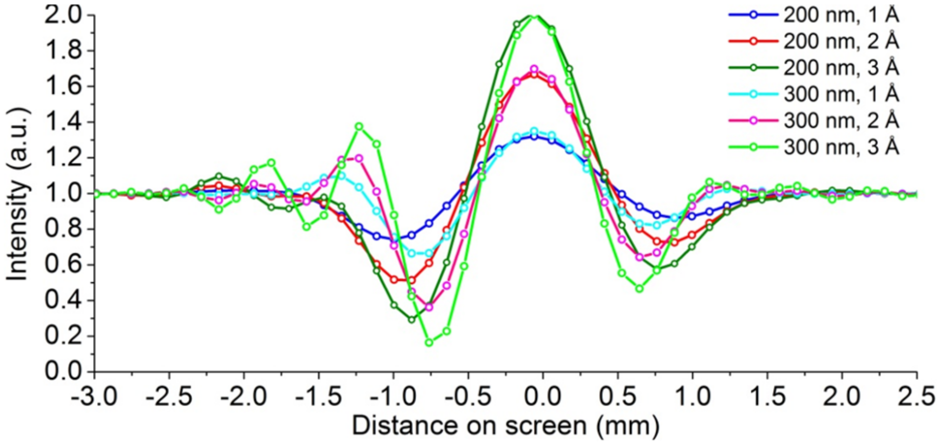

Supplementary Figure 2. Intensity profiles through the centre of the (-1010) divergent beam electron diffraction (DBED) spot at different simulation parameters. Ripple amplitude is 1, 2 and 3 Å, and the source-to-sample distance is 200 nm and 300 nm. In these simulations, the source-to-detector distance is 50 mm, and the electron energy is 230 eV. It can be seen that the ripple with higher amplitude results in an increased intensity contrast, which can be intuitively expected. Also, the same ripple can produce different contrast at different source-to-sample distances: at larger source-to-sample distance, the width of the intensity distribution in the first-order diffraction spot is de-magnified due to the decreased magnification, but the contrast becomes higher.

Supplementary Note 1

Imaging weak phase objects

The transmission function of a weak phase object can be approximated as:

$$t(x,y) = e^{i\Delta\varphi(x,y)} \approx 1 + i\Delta\varphi(x,y), \qquad (1)$$

where (x,y) are the coordinates in the object plane and $\Delta\varphi(x,y)$ is the phase shift introduced to the incident wave. Since a constant phase shift can be added to a wave without changing its intensity distribution, the phase distribution superimposed onto the incident wave $\Delta\varphi(x,y)$ can be re-written so that $\Delta\varphi(x,y) > 0$. Equation (1) allows for the splitting of

the exit wave into two terms: the number 1 describes the reference wave and $i\Delta\varphi(x,y)$ describes the perturbation to the reference wave caused by the object and thus can be interpreted as the object wave. Because the phase change superimposed onto the incident wave in the object domain is preserved while the wave propagates toward the detector we can write

$$L\left(1+i\Delta\varphi(x,y)\right)\rightarrow 1+i\Delta\varphi(X,Y), \tag{2}$$

where L is an operator of forward propagation towards the detector plane, and (X,Y) are the coordinates in the detector plane. The intensity on the detector is given by:

$$I(X,Y)=\left|1+i\Delta\varphi(\mathrm{X},Y)\right|^2=1+\left|\Delta\varphi(X,Y)\right|^2. \tag{3}$$

Equation (3) describes how the three-dimensional surface of a ripple is transformed into the contrast of the intensity distribution. For a ripple with low amplitude h, the approximation of weak phase shift applies and Eq. (3) can predict the intensity contrast caused by the ripple:

$$\Delta I(X,Y)=\left|\Delta\varphi(X,Y)\right|^2\approx\left(\frac{2\pi}{\lambda}h(1-\cos\vartheta)\right)^2.$$
